# What Is the Role of Allogeneic Cortical Strut Grafts in the Treatment of Fibrous Dysplasia of the Proximal Femur?

**DOI:** 10.1007/s11999-016-4806-3

**Published:** 2016-03-28

**Authors:** Bastiaan C. J. Majoor, Marleen J. M. Peeters-Boef, Michiel A. J. van de Sande, Natasha M. Appelman-Dijkstra, Neveen A. T. Hamdy, P. D. S. Dijkstra

**Affiliations:** 1 Department of Orthopaedic Surgery, Leiden University Medical Center, Centre for Bone Quality, Potzone J11R, Postbus 9600, 2300 RD Leiden, The Netherlands; 2 Department of Medicine, Division of Endocrinology, Leiden University Medical Center, Centre for Bone Quality, Leiden, The Netherlands

## Abstract

**Background:**

Management of fibrous dysplasia of the proximal femur is a progressive, often recurrent condition of bone that can cause skeletal deformity, fractures, and pain. Allogeneic cortical strut grafting to minimize the risk of fracture or as part of fracture treatment is a promising treatment option, but evidence is scarce on the intermediate- to long-term results of this procedure and there are no data on factors associated with graft failure.

**Questions/purposes:**

The purposes of this study were (1) to evaluate the revision-free survivorship; (2) radiographic findings; (3) factors associated with failure; and (4) complications associated with cortical strut allograft to prevent or treat fractures of the proximal femur in patients with fibrous dysplasia.

**Methods:**

Between 1980 and 2013 we performed cortical strut allografting in 30 patients for impending or actual fractures of the proximal femur, of whom 28 (93%) were available for followup at a minimum of 2 years (mean, 13 years; range, 4–37 years) and of whom 22 (73%) had also been evaluated within the last 5 years. During that time, the indications for cortical strut allografting were an impending fracture of the proximal femur, persistent pain, or an actual nondisplaced femoral fracture. In patients who presented with a diaphyseal fracture, a fracture with severe dislocation of severe varus deformity, which required an osteotomy, placement of a blade plate was instead performed and these patients are not included here. During that time, for patients with diaphyseal fractures, and in patients with a displaced femoral fracture of the proximal femur, placement of a blade plate without strut grafting was instead performed; these patients are not included here. The primary outcome was the success rate of allogeneic cortical strut grafting surgery as assessed by the absence of revision surgery for a newly sustained fracture, resorption of the graft, or progressive deformity of the proximal femur. The association of possible contributing factors to graft failure such as gender, age at surgery, preoperative fracture, and anchoring distances of the graft in healthy bone was also evaluated using Cox regression analysis.

**Results:**

Revision surgery was performed in 13 patients, resulting in a mean survival time of 13 years (Kaplan-Meier 95% confidence interval [CI], 10–16). Radiological resorption of the graft was observed in 15 of 28 patients (54%). However, revision surgery was not performed in all patients who developed graft resorption, because of the absence of a risk for fracture on the basis of the anatomical site of resorption. Identified risk factors for graft failure included preoperative fractures (hazard ratio [HR], 4.5; 95% CI, 1.2–17.2; p = 0.028) and insufficient proximal anchoring of the graft in healthy bone (HR, 6.02; 95% CI, 1.3–27; p = 0.02). One patient sustained a refracture after surgery resulting from an in-hospital fall. The fracture was treated without further surgery, and it healed.

**Conclusions:**

Our findings from this study suggest that cortical strut allografting may be a viable option for treatment of fibrous dysplasia of the proximal femur a without previous pathological fracture. Surgeons should pay particular attention to the proximal fixation point of the allograft to decrease the risk of failure. Patients with a fracture have an increased risk of failure and reoperation and so should be treated with an osteosynthesis.

**Level of Evidence:**

Level IV, therapeutic study.

## Introduction

Fibrous dysplasia (fibrous dysplasia) is a rare benign bone disease caused by a postzygotic, activating mutation of the GNAS gene, which alters the signaling of G-protein at the cellular level. The bone lesions are characterized by local replacement of healthy bone by fibrous tissue, which is produced by poorly differentiated osteoblasts, osteoclast activation, and local increase in bone turnover. Clinical manifestations include pain, deformities, and increased risk for fractures. The spectrum of fibrous dysplasia includes single lesions (monostotic fibrous dysplasia), multiple lesions (polyostotic fibrous dysplasia), and the combination of polyostotic disease with extraskeletal manifestations such as precocious puberty, hormonal dysregulation, and café-au-lait skin patches as observed in McCune-Albright syndrome. Although lesions may occur in any bone, the proximal femur and craniofacial bones are the predominant localizations of fibrous dysplasia [3]. As a result of the weightbearing properties of the proximal femur, lesions at this site are vulnerable to microfractures, which may be associated with pain, pathological fractures, and ultimately a varus deformity of the femoral neck, leading to the “shepherd’s crook deformity” characteristic of fibrous dysplasia lesions at this site.

Lesions of the proximal femur historically have been treated with curettage and cancellous bone grafting [10]. However, these procedures were associated with a high risk of local recurrence, and the use of cortical grafts subsequently was proposed as a preferable alternative on the basis that cortical bone may be less prone to replacement by dysplastic tissue [7, 9]. In 2005 DiCaprio and Enneking [6] suggested that allogeneic cortical strut grafting should be used instead of autogenous cortical bone in fibrous dysplasia because they would be less likely or at least slower to be replaced by dysplastic tissue, therefore providing better material for grafting.

Whereas failure rates were reported to be lower in allogeneic cortical strut grafting compared with cancellous bone grafting, it has so far been difficult to anticipate which patients are more likely to benefit from allogeneic cortical strut grafting and which factors are associated with graft failure [6, 10]. In addition, to our knowledge, there are few reports on long-term followup of patients treated with cortical strut allografting; because fibrous dysplasia has a propensity to recur, this is an important gap in knowledge.

We therefore sought (1) to evaluate the revision-free survivorship; (2) radiographic findings; (3) factors associated with failure; and (4) complications associated with cortical strut allograft to prevent or treat fractures of the proximal femur in patients with fibrous dysplasia.

## Patients and Methods

Data on all patients who received an allogeneic cortical strut graft for fibrous dysplasia of the proximal femur from 1980 to 2013 at the Orthopaedic Department of the Leiden University Medical Center were evaluated in a retrospective study design. In The Netherlands, this kind of research does not need approval of the ethical committee.

### Patient Population

Between 1980 and 2013 we performed cortical strut allografting in 34 patients for impending or actual fractures of the proximal femur or for persistent pain nonresponsive to medical treatment. Patients who underwent additional valgus osteotomy (n = 4) were excluded from the study, because the aim of our study was to evaluate the efficacy of allogeneic cortical strut grafting in preventing varus deformity rather than to correcting it. Another two patients were excluded because followup was below the minimum of 2 years. This left 28 patients (82%) available for followup at a minimum of 2 years (mean, 13 years; range, 4–37 years), of whom 22 (73%) had also been evaluated within the last 5 years.

Sixteen of the 28 patients studied (57%) had monostotic disease, 11 (39%) had polyostotic disease, and one patient had McCune-Albright syndrome (Table 1). Gender was evenly distributed (15 female, 13 male). Median age at the time of allogeneic cortical strut grafting was 23 years (range, 5–50 years), and mean followup after surgery was 13 years (range, 4–37 years). Four patients had surgery of the proximal femur before allogeneic cortical strut graft surgery and 11 patients had a preoperative fracture (for details, see Table 1). Of the 28 patients who were treated with allogeneic cortical strut grafting, 27 received a fibular strut graft and one patient received a tibial strut graft (seven dual struts and 21 single). Twenty-one patients were additionally treated with curettage and placement of allogeneic cancellous bone during the allogeneic cortical strut grafting procedure.

**Table 1:** Patient characteristics

Patient number	Gender	Type of FD	Age at surgery (years)	Prior surgery	Preoperative fracture	Type of graft	Curettage + cancellous graft	Number of struts	Proximal anchoring ratio	Failure mechanism	Reoperation	Followup (years)
1	F	Polyostotic	48	CBG	No	Fibula	No	1	13%	–	No	19
2	F	Monostotic	31	No	No	Fibula	No	1	19%	–	No	4
3	F	Monostotic	39	No	No	Fibula	Yes	1	–	–	No	20
4	M	Polyostotic	10	No	No	Tibia	No	2	3%	Resorption	Yes	26
5	M	Polyostotic	12	No	No	Fibula	No	1	9%	–	No	25
6	M	Monostotic	10	No	Yes	Fibula	No	1	3%	Fracture	Yes	14
7	F	Monostotic	27	CBG	Yes	Fibula	Yes	2	3%	Resorption	Yes	28
8	M	Monostotic	37	No	No	Fibula	Yes	1	10%		No	7
9	M	Monostotic	12	No	Yes	Fibula	Yes	1	7%	Resorption	Yes	5
10	F	Polyostotic	17	No	Yes	Fibula	Yes	2	3%	Resorption	Yes	12
11	F	Polyostotic	45	No	No	Fibula	Yes	1	8%	–	No	7
12	M	Polyostotic	5	No	Yes	Fibula	Yes	1	3%	Fracture	Yes	9
13	F	Polyostotic	22	No	Yes	Fibula	Yes	1	4%	Deformity	Yes	25
14	M	Monostotic	24	No	No	Fibula	Yes	1	6%	–	No	6
15	F	Monostotic	23	CBG	Yes	Fibula	Yes	2	3%	Fracture	Yes	9
16	F	Polyostotic	27	No	No	Fibula	Yes	1	17%	–	No	6
17	F	Monostotic	15	No	No	Fibula	Yes	2	20%	–	No	6
18	M	Monostotic	8	No	Yes	Fibula	Yes	1	6%	–	No	4
19	M	Polyostotic	27	No	No	Fibula	Yes	1	5%	Resorption	Yes	25
20	M	Monostotic	9	No	No	Fibula	Yes	1	10%	–	No	11
21	F	Monostotic	18	No	No	Fibula	Yes	2	6%	Fracture	Yes	4
22	M	Monostotic	50	No	No	Fibula	Yes	1	8%	–	No	11
23	F	Monostotic	21	No	No	Fibula	Yes	1	17%	–	No	8
24	M	Monostotic	24	No	No	Fibula	Yes	2	4%	Resorption	Yes	7
25	F	MAS	14	Osteosynthesis	Yes	Fibula	Yes	1	8%	–	No	37
26	F	Polyostotic	44	No	No	Fibula	No	1	19%	–	No	10
27	F	Monostotic	10	No	Yes	Fibula	No	1	5%	Resorption	Yes	5
28	M	Polyostotic	25	No	Yes	Fibula	Yes	1	1%	Resorption	Yes	13

FD = fibrous dysplasia; F = female; M = male; CBG = cancellous bone grafting; MAS = McCune-Albright syndrome.

### Treatment Algorithm

**Fig. 1:**
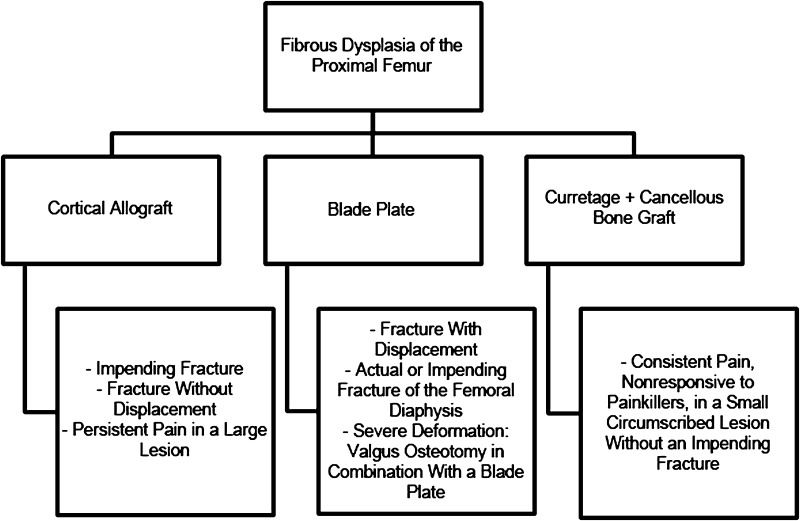
The protocol for surgical treatment of fibrous dysplasia of the proximal femur in our center is shown.

The indications (Fig. 1) for cortical strut allografting during the followup period were agreed on by all participating surgeons. They included impending fracture of the proximal femur, persistent pain, or an actual nondisplaced femoral fracture. During that time, for patients with diaphyseal fractures, patients who were treated with an osteotomy and in patients with a displaced fracture of the proximal femur, placement of a blade plate without strut grafting were instead performed (nine patients); these patients are not included in the current study. Curettage in combination with cancellous bone grafting was performed occasionally (five patients) in individuals presenting with pain but with a small, circumscribed lesion and whose images did not suggest a risk of pathological fracture; likewise, those patients are not included in this study.

### Allogeneic Cortical Strut Grafting Technique

The patient was placed in a supine position and a straight lateral incision was made to expose the greater trochanter and diaphysis; the femur was reached posteriorly to the vastus lateralis. A Kirschner wire was introduced in the fibrous dysplasia lesion under guidance of fluoroscopy by passing it through the lateral cortical bone at the level of the lesser trochanter, pushing it through the fibrous dysplasia lesion to the end in the femoral head, specifically aiming for the tip of the Kirschner wire ending in vital cancellous bone while evading the physis if still open. A cannulated reamer was then placed over the Kirschner wire to create a fitting tunnel for the allogeneic cortical strut grafting. Material was obtained from the lesion if biopsy had not been performed before the procedure. Curettage of the defect was not routinely performed but particularly in cases with scalloping and thinning of the cortex in which case it was necessary to partially fill the lesion with cancellous bone graft. The diameter of the strut graft was compared with the drilled tunnel to secure smooth insertion of the allogeneic cortical strut grafting. Under fluoroscopy a Kirschner wire was introduced into the center of the allogeneic cortical strut grafting for more accurate docking. The cortical allograft was then placed over the Kirschner wire and the lateral protruding graft was leveled with the femoral cortex. An additional allogeneic cortical strut graft was inserted in lesions that involved more than three-fourths the diameter of the femoral neck.

Patients were encouraged to mobilize postoperatively using two crutches and partial weightbearing (up to a maximum of 15 kg). Gradual increase in weightbearing was allowed after 6 weeks if increasing consolidation of the graft was observed on plain radiographs.

### Outcomes Assessment

The primary outcome of our study was the proportion of patients undergoing revision surgery as a result of fracture, progressive deformity, and/or progressive resorption of the graft with return of pain.

Resorption of the graft was determined by evaluation of consecutive, yearly radiographs undertaken by one of the authors (BCJM). Grafts were scored as “totally resorbed” if over 50% of the graft was resorbed or if resorption extended to the full diameter of the graft.

**Fig. 2:**
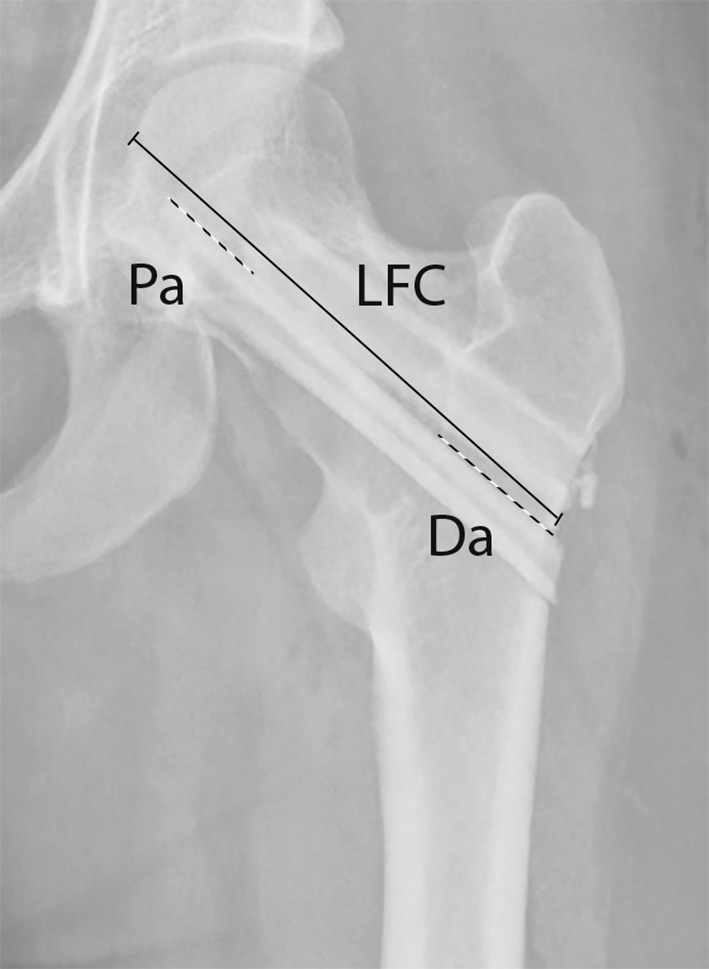
Assessment of the anchoring ratio of the graft in vital bone. The proximal anchoring (Pa) and distal anchoring (Da) parts of the graft were measured and divided by the length of the femoral collum (LFC) to obtain the ratio. In case two grafts were used, we chose the measurement with the deepest anchoring. LFC was defined by as the length between the lateral cortex and femoral head in alignment with the graft.

Potential risk factors for revision surgery were also assessed, including gender, age at the time of surgery, a preoperative fracture, proximal and distal anchoring of the graft in healthy bone, concurrent curettage of the fibrous dysplasia lesion during allogeneic cortical strut grafting surgery, and concurrent placement of cancellous bone during allogeneic cortical strut grafting surgery. Proximal and distal anchoring was assessed by measuring the length of both the proximal and distal parts of the graft that were anchored in vital bone (Pa and Da in Fig. 2) and the length of the femoral neck (LFC in Fig. 2). We then calculated the ratio of the proximal and of the distal length of the graft in vital bone to the length of the femoral neck. The ratio had to be used because old radiographs were used without calibrated measuring options.

### Statistical Analysis

Statistical analysis was performed with the use of SPSS for Windows, Version 23.0 (SPSS, Inc, Chicago, IL, USA). Survival analysis was performed with the use of the Kaplan-Meier method. Risk factors were assessed with use of the log-rank test and/or with a univariate Cox regression model and results are presented as mean ± SD. The influence of continuous data, for example age at the time of surgery, was analyzed with the use of a linear regression model.

## Results

### Revision-free Survivorship

**Fig. 3:**
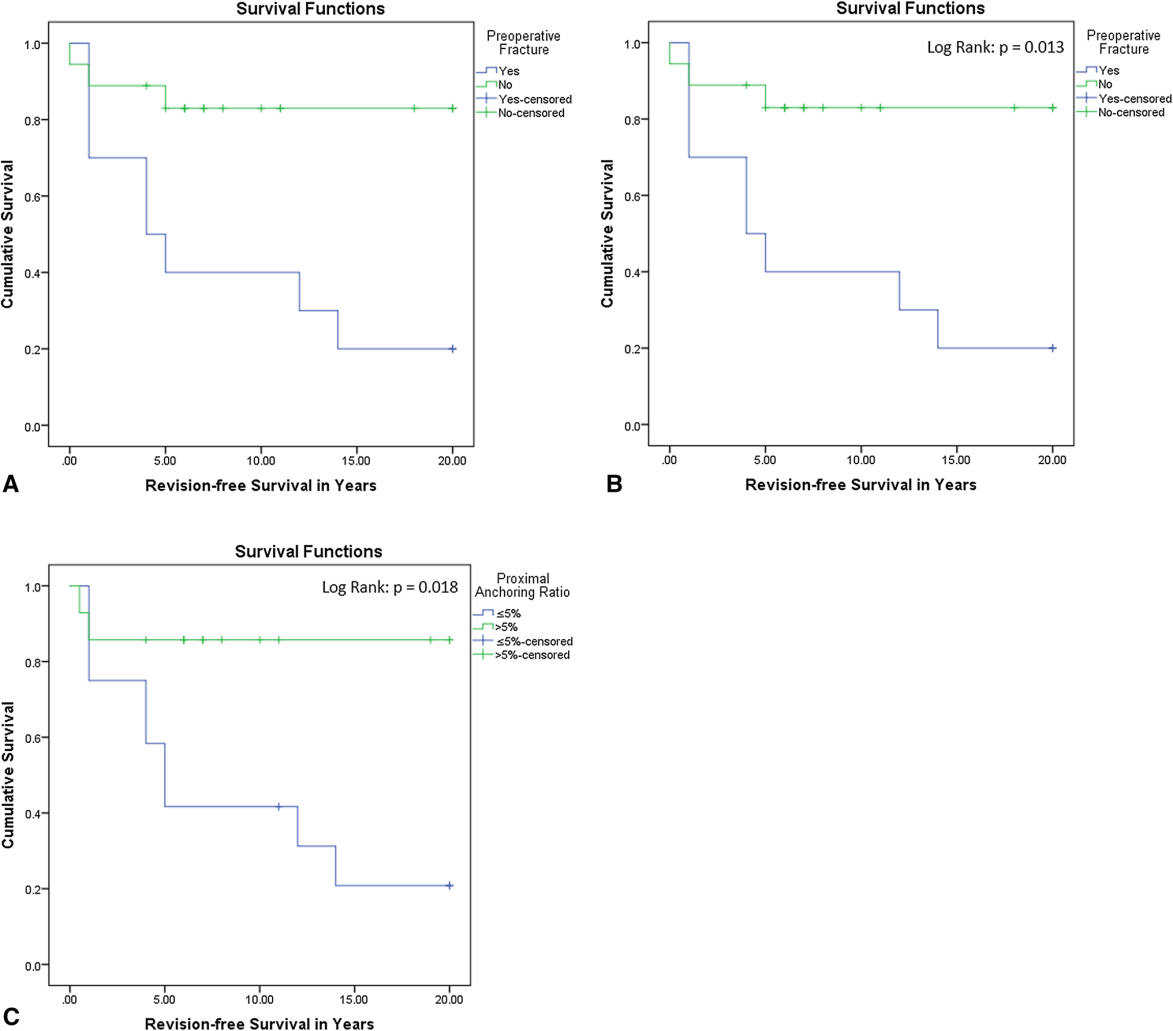
**A**–**C** The Kaplan-Meier curve for revision-free survival (**A**) indicates that most failures occur in the first 5 years after surgery. The Kaplan-Meier curves (**B**–**C**) illustrate the role of a preoperative fracture and insufficient proximal anchoring on revision-free survival.

Overall revision-free survival was 54% after 20 years and mean survival time in Kaplan-Meier (Fig. 3) was 13 years (95% confidence interval [CI], 10–16). Thirteen of 28 patients (46%) underwent a reoperation as a result of resorption of the graft (61%), a fracture (31%), or as a result of progressive deformation of the proximal femur (8%). Mean time to graft failure was 7 ± 8 years.

### Radiological Appearance of Grafts

**Fig. 4:**
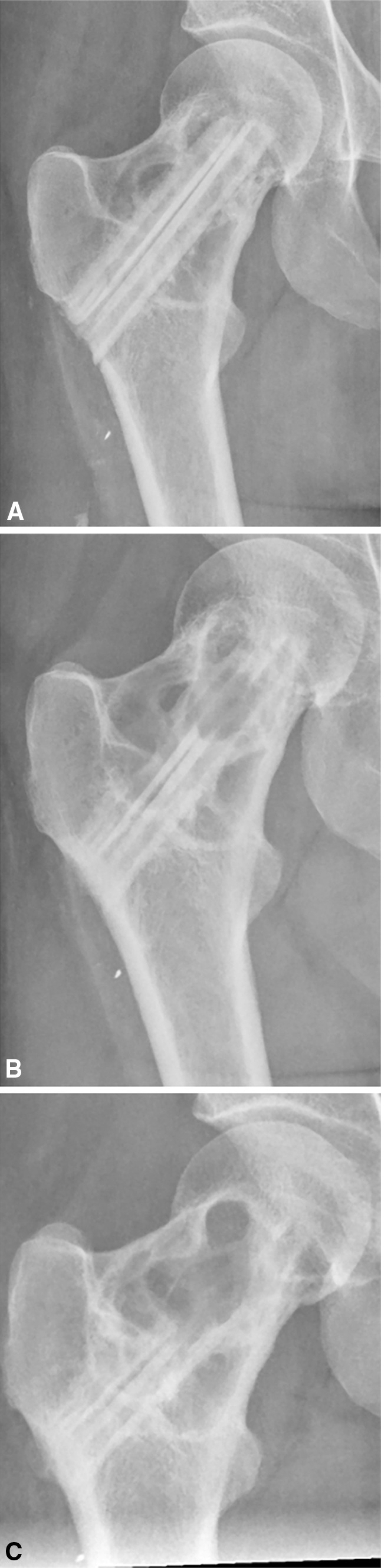
**A**–**C** The radiograph (**A**) is made postoperatively and shows two fibular strut grafts that cross the dysplastic lesion but have minimal contact with vital bone proximally. After 1 year, resorption of the graft gradually increased (**B**) and finally the strut graft is resorbed over the full length of the diameter (**C**), losing its stabilizing function.

**Fig. 5:**
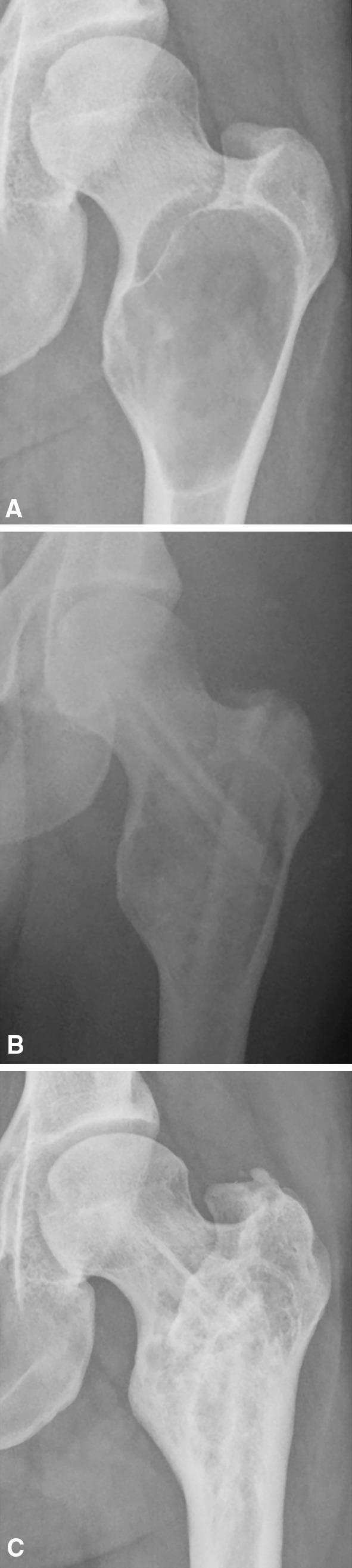
**A**–**C** The radiograph of Patient 17 shows an expansive lesion in the proximal femur with a ground glass aspect and cortical thinning (**A**). The diagnosis of fibrous dysplasia was histologically confirmed and the patient was treated with implantation of two fibular strut grafts (**B**). The strut grafts gradually incorporated in vital bone and the radiograph showed a stable situation 7 years after surgery. (**C**) The patient could be discharged from further controls with good function and no pain.

Radiological resorption of the graft (Fig. 4) was observed in 15 patients (54%). However, revision surgery was not performed in all patients who developed graft resorption, because according to the treatment protocol in our center, surgery is only required in case of an impending or actual fracture and/or persistent pain. The other 13 patients showed full incorporation of the bone graft (Fig. 5).

### Factors Associated With Survivorship of Grafts

Preoperative fracture was associated with increased risk for revision surgery (hazard ratio [HR], 4.5; 95% CI, 1.2–17.2; p = 0.028; Table 2) as was insufficient proximal anchoring of the graft in vital bone (HR, 6.0; 95% CI, 1.3–27.0; p = 0.020), although this was not the case for insufficient distal anchoring (HR, 1.4; 95% CI, 0.5–4.2; p = 0.543) with the numbers available. Revision-free survival curves of the risk factors confirmed the results of the Cox regression analysis (Fig. 3). Gender, type of fibrous dysplasia, additional curettage and/or cancellous bone grafting, and previous surgery before allogeneic cortical strut graft surgery did not appear to be associated with reoperation after allogeneic cortical strut graft surgery with the numbers available. Although patients who received cortical allografts at a young age appeared to have an increased risk of reoperation compared with older patients, age was not associated with revision surgery (HR, 1.05/year; 95% CI, 1.0–1.1; p = 0.087).

**Table 2:** Risk factors for failure of ACSG surgery (univariate Cox regression analysis)

Risk factor	Hazard ratio	95% confidence interval	p value
Monostotic fibrous dysplasia	0.50	0.2–1.6	0.230
Previous surgery	0.79	0.2–3.6	0.756
Additional curettage + CBG	1.34	0.4–4.9	0.657
Distal anchoring ratio ≤ 5%	1.40	0.5–4.2	0.543
Gender (male)	1.40	0.5–4.2	0.543
Preoperative fracture	4.50	1.2–17.2	0.028
Proximal anchoring ratio ≤ 5%	6.02	1.3–27.0	0.020
Age at surgery	1.05/year	1.0–1.1	0.087

ACSG = allogeneic cortical strut graft; CBG = cancellous bone grafting.

### Complications

One patient sustained a refracture after surgery resulting from an in-hospital fall. The fracture was treated without further surgery, and it healed. No other complications of surgery were observed.

## Discussion

Despite progress in understanding the pathogenesis of fibrous dysplasia, its treatment has been subject to controversy ever since the first reports of Lichtenstein on management of the disease, emphasizing its notorious recurrent character, the wide variations in phenotype, and the lack of a successful treatment strategy [15, 16]. Over the past few decades many surgical approaches have been proposed and discarded in the management of fibrous dysplasia ranging from particular forms of grafting to a variety of types of implants or a combination of both [6]. Although curettage and bone grafting using cancellous bone have been historically the treatments of choice, the perspective of the value of this treatment in fibrous dysplasia lesions of the proximal femur has altered over the last decades as a result of increasing reports on the marginal outcome of the procedure (Table 3) [10–12, 18, 19, 23]. We theorized that allogeneic cortical strut grafting might improve the outcome of fibrous dysplasia lesions of the proximal femur, arguing that cortical allografts would be less prone to be affected by pathological fibrous dysplasia bone and therefore less prone to resorb and fail. We found that allogeneic cortical strut grafting has a survivorship of 54% after long-term followup and that patients who presented with fracture (as opposed to impending fracture) and patients whose grafts lacked sufficient proximal fixation were at increased risk of undergoing reoperation.

**Table 3:** Previous studies into surgical treatment of fibrous dysplasia of the femoral neck

Study	Number	Type of graft	Mean followup	Failure graft	Clinical outcome
Harris et al. (1962) [11]	10	Cancellous autograft	Unknown	5/10	Five of 10 had a poor outcome
Nakashima et al. (1984) [18]	8	Autograft	Unknown	2/8	25% had a poor outcome
Enneking and Gearen (1986) [7]	15	Cortical autograft	6 years	2/15	Two of 15 had a poor outcome (reoperation)
Stephenson et al. (1987) [23]	18	Cancellous autograft	10.4 years	25/31	81% had a poor outcome
Guille et al. (1998) [10]	22	Cancellous autograft	15 years	22/22	100% had resorption
Ippolito et al. (2003) [12]	5	Cancellous autograft	Unknown	3/5	Three of 5 patients had a poor outcome (reoperation)
George et al. (2008) [9]	8	Cortical autografts	4.1 years	1/8	One patient had a poor outcome (recurrence).
Tong et al. (2013) [24]	15	Cancellous autograft with internal fixation	12–32 months	0/15	No patients needed a reoperation
Nishida et al. (2015) [19]	8	Cortical autograft with internal fixation	75 months	0/8	No patient had a poor outcome

This study had a number of limitations. First, the small number of patients included in our study reflects the low prevalence of symptomatic fibrous dysplasia, although our series of patients was larger than any reported thus far of which we are aware. Because of a small study size, it is possible that we were unable to detect the less common complications of allogeneic cortical bone grafting. Furthermore, we have to take into account the possibility that some of the risk factors that were not associated with treatment outcome in our study might show an association in larger studies. Second, the long span of time over which this retrospective study’s procedures were performed saw many changes in patient care. Although the indications were generally consistent over time at the study site, it is impossible to know with certainty that they were applied with precision over the nearly 35-year timeframe. Also, many of these procedures were performed before patient-reported outcomes tools came into wide use, and so we could not report patient-reported outcomes here. It is our impression based on chart review and patient surveys done after surgery that pain improved in most of these patients. Furthermore, we appreciate that the variability in treatment options for fibrous dysplasia of the proximal femur, which is no doubt the result of the heterogeneity of the disease, makes it difficult to compare different patients. Although the use of multiple trajectories, additional cancellous bone grafting, and additional curettage were taken into account in our analysis, these different approaches within the treatment with cortical allografts might affect the outcome in larger studies. Finally, although the treatment of fibrous dysplasia is centralized in The Netherlands, it is common in long-term studies that some patients were lost to followup.

Although a large proportion of patients in this study (nearly half of them) underwent revision at some point during the followup period, we consider the fact that more than half of the patients did not undergo further surgery actually a reasonably good result. The reason for this is that the condition recurs so commonly, and other described approaches actually reported even more frequent failures than we observed here [10, 23]. Most previous studies were restricted to limited followup and are therefore likely to overestimate the therapeutic effect of bone grafting, a fact that is further emphasized by studies with long-term followup generally, suggesting a poorer outcome (Table 3). Because of its mean followup of 13 years, our study gives a fair representation of the long-term effects of allogeneic cortical strut graft treatment in fibrous dysplasia lesions of the proximal femur. However, Kaplan-Meier survivorship analysis (Fig. 3A) showed that most reoperations were performed within 5 years after the primary surgery, indicating that after 5 years, failure of allogenous cortical strut grafts leading to reoperation is less likely to be expected. In addition, besides being biologically preferable to prevent graft resorption, the use of allogeneic bone has the advantage of no additional surgery being required to retrieve autogenous bone.

Slightly more than half of our patients experienced radiographic evidence of graft resorption. Again, however, we consider this a reasonably good result considering the problems reported using other techniques when dealing with proximal femoral lesions in patients with fibrous dysplasia, especially in studies addressing cancellous bone grafting in which recurrence is reported in nearly all patients [10, 23]. Although the indication for reoperation was graft resorption in the majority of the cases (61%), graft resorption was not an indication for reoperation per se, because we would only perform a reoperation in case graft resorption led to an impending or an actual fracture.

We identified several risk factors for failure of allogeneic cortical strut graft surgery in this study. In patients undergoing cortical strut allografting, we found a minimal proximal anchoring ratio in vital bone of 5% is required to enable the graft to be incorporated in the proximal femur. Therefore, proper evaluation of proximal placement preoperatively and intraoperatively should be mandatory, whereas this is not so for distal anchoring in which case anchoring in the cortex will generally suffice. Although it has previously been reported that insufficient proximal docking in healthy bone might have played a role in failure of allogeneic cortical strut grafting in two patients in the Enneking/Gearen study [7], our study is the first to clearly identify insufficient proximal anchoring of the graft and preoperative fractures as risk factors for failure of allogeneic cortical strut graft surgery.

Our data also demonstrate a higher risk of revision in patients with a preoperative fracture. Patients who sustained a fracture of the proximal femur at some point before surgery have thus a high risk of failure of allogeneic cortical strut grafting by either resorption or a consecutive fracture. We suspect that a pathological fracture is only the endpoint of a sliding scale. Guille et al. already identified involvement of the calcar femorale as a risk factor for failure [10]. If the calcar is involved, the proximal femur will considerably lose stability by loss of redistribution of stress forces [26]. Subsequently the proximal femur will be more prone to fractures. A cortical strut graft will very likely not be able to address the extensive forces applied on the proximal femur without this form of stability. Based on the results with internal fixation in other studies [1, 4, 5, 8, 10, 12–14, 17, 20, 23–25] and the findings from our present study, we recommend primary internal fixation in patients with extensive lesions that threaten to fracture or have already induced a fracture, because this approach has shown promising results in several studies. More importantly, although only the combination of internal fixation and autografts has so far been studied, there might be an important role for implantations in combination with cortical allografts in patients with preoperative risk factors. Although the polyostotic form of fibrous dysplasia is generally associated with a worse outcome compared with monostotic disease [6, 10], we were not able to identify polyostotic fibrous dysplasia as a risk factor for graft failure. This may be explained by the fact that polyostotic fibrous dysplasia can be profoundly variable in its course and in the extent of lesions. Patients with extensive lesions, both monostotic and polyostotic fibrous dysplasia, were primarily treated with internal fixation, because a strut graft would not properly bridge the lesion and therefore local expansion and not the type of fibrous dysplasia would be a risk factor. Furthermore, we expected that young age at the time of surgery would be a risk factor based on the hypothesis that fibrous dysplasia tends to be more active and aggressive during childhood and because patients with extensive disease are generally diagnosed at a younger age [22]. However, we were unable to demonstrate this in the present study, perhaps because of the statistical limitations imposed on our statistical analysis by the sample size.

Although this procedure can be technically challenging, we were gratified by the relative rarity of complications in this series. This compares favorably to bone grafting with autogenous bone, which can be accompanied by complications at the donor site [2, 21].

Our findings from this study suggest that cortical strut allografting may be a viable option for treatment of fibrous dysplasia of the proximal femur who have not already experienced a fracture. Surgeons should pay particular attention to the proximal fixation point of the allograft to decrease the risk of failure. Patients with a fracture have an increased risk of failure and reoperation and so should be treated with osteosynthesis.
